# Incremental Velocity Error as a New Treatment in Vestibular Rehabilitation (INVENT VPT) Trial: study protocol for a randomized controlled crossover trial

**DOI:** 10.1186/s13063-021-05876-4

**Published:** 2021-12-11

**Authors:** Ann-Margret Ervin, Michael C. Schubert, Americo A. Migliaccio, Jamie Perin, Hamadou Coulibaly, Jennifer L. Millar, Dale Roberts, Mark Shelhamer, Daniel Gold, Stephanie Beauregard, Robin Pinto, Douglas Brungart, Bryan K. Ward, for the INVENT VPT Research Group

**Affiliations:** 1 Johns Hopkins Bloomberg School of Public Health, 615 N. Wolfe Street, Baltimore, MD 21205 USA; 2 Johns Hopkins University School of Medicine, 601 N. Caroline Street, Baltimore, MD 21287 USA; 3 Neuroscience Research Australia, Sydney, Australia; 4 Johns Hopkins University School of Medicine, 1800 Orleans Street, Baltimore, MD 21287 USA; 5 Intrepid Spirit Center, Fort Belvoir Community Hospital, Intrepid Pavilion, 5980 9th Street, Bldg. 1259, Fort Belvoir, VA 22060 USA; 6 National Military Audiology and Speech Center, Walter Reed National Military Medical Center, Building 19, Room 5600, 4954 North Palmer Rd, Bethesda, MD 20889-5630 USA

**Keywords:** Vestibular rehabilitation, Vestibulo-ocular reflex, Dizziness, Incremental vestibular adaptation, Imbalance, Service members, Traumatic brain injury

## Abstract

**Background:**

A clinical pattern of damage to the auditory, visual, and vestibular sensorimotor systems, known as multi-sensory impairment, affects roughly 2% of the US population each year. Within the population of US military service members exposed to mild traumatic brain injury (mTBI), 15–44% will develop multi-sensory impairment following a mild traumatic brain injury. In the US civilian population, multi-sensory impairment-related symptoms are also a common sequela of damage to the vestibular system and affect ~ 300–500/100,000 population. Vestibular rehabilitation is recognized as a critical component of the management of multi-sensory impairment. Unfortunately, the current clinical practice guidelines for the delivery of vestibular rehabilitation are not evidence-based and primarily rely on expert opinion. The focus of this trial is gaze stability training, which represents the unique component of vestibular rehabilitation. The aim of the Incremental Velocity Error as a New Treatment in Vestibular Rehabilitation (INVENT VPT) trial is to assess the efficacy of a non-invasive, incremental vestibular adaptation training device for normalizing the response of the vestibulo-ocular reflex.

**Methods:**

The INVENT VPT Trial is a multi-center randomized controlled crossover trial in which military service members with mTBI and civilian patients with vestibular hypofunction are randomized to begin traditional vestibular rehabilitation or incremental vestibular adaptation and then cross over to the alternate intervention after a prescribed washout period. Vestibulo-ocular reflex function and other functional outcomes are measured to identify the best means to improve the delivery of vestibular rehabilitation. We incorporate ecologically valid outcome measures that address the common symptoms experienced in those with vestibular pathology and multi-sensory impairment.

**Discussion:**

The INVENT VPT Trial will directly impact the health care delivery of vestibular rehabilitation in patients suffering from multi-sensory impairment in three critical ways: (1) compare optimized traditional methods of vestibular rehabilitation to a novel device that is hypothesized to improve vestibulo-ocular reflex performance, (2) isolate the ideal dosing of vestibular rehabilitation considering patient burden and compliance rates, and (3) examine whether recovery of the vestibulo-ocular reflex can be predicted in participants with vestibular symptoms.

**Trial registration:**

ClinicalTrials.gov NCT03846830. Registered on 20 February 2019.

## Administrative information

Note: the numbers in curly brackets in this protocol refer to SPIRIT checklist item numbers. The order of the items has been modified to group similar items (see https://www.spirit-statement.org/).

**Table:** 

Title {1}	Incremental Velocity Error as a New Treatment in Vestibular Rehabilitation (INVENT VPT Trial): study protocol for a randomized controlled crossover trial
Trial registration {2a and 2b}.	ClinicalTrials.gov NCT03846830. Registered on 20 February 2019 Incremental Velocity Error as a New Treatment in Vestibular Rehabilitation (INVENT VPT Trial).
Protocol version {3}	Version 1.0, 15 October 2020
Funding {4}	This work was supported by the Department of Defense under the Psychological Health/Traumatic Brain Injury Research Program Complex Traumatic Brain Injury Rehabilitation Research Clinical Trial Award (Grant Award W8lXWH-l7).
Author details {5a}	*Ann-Margret Ervin*^1^*, Michael C. Schubert*^*2-4*^*, Americo A. Migliaccio*^*5*^*, Jamie Perin*^6^*, Hamadou Coulibaly*^1^*, Jennifer L. Millar*^*2,4*^*, Dale Roberts*^*7*^*, Mark Shelhamer*^*2,3*^*, Daniel Gold*^*7*^*, Stephanie Beauregard*^*8*^*, Robin Pinto*^*9*^*, Douglas Brungart*^*9*^*, and Bryan K. Ward*^*3*^ *for the INVENT VPT Research Group* ^1^Johns Hopkins Bloomberg School of Public Health, Department of Epidemiology, Baltimore, MD, United States of America ^2^Laboratory of Vestibular NeuroAdaptation, Department of Otolaryngology - Head and Neck Surgery, Johns Hopkins University, Baltimore, MD, United States of America ^3^ Department of Otolaryngology - Head and Neck Surgery, Johns Hopkins University, Baltimore, MD, United States of America ^4^ Department of Physical Medicine and Rehabilitation, Johns Hopkins University, Baltimore, MD, United States of America ^5^ Neuroscience Research Australia, Sydney, Australia ^6^ Johns Hopkins Bloomberg School of Public Health, Department of International Health, Baltimore, MD, United States of America ^*7*^Department of Neurology, Johns Hopkins University School of Medicine, Baltimore, MD, United States of America ^8^Fort Belvoir Community Hospital, Fort Belvoir Virginia, United States of America ^9^Walter Reed National Military Medical Hospital, Rockville MD, United States of America MCS conceived the study. All authors initiated the study design and assisted the implementation of the trial. MCS is the grant holder. AAM designed and built the rehabilitation devices. AE, JP, and HC provided expertise in clinical trial design and JP is conducting the primary statistical analysis. All authors contributed to refinement of the study protocol and approved the final manuscript.
Name and contact information for the trial sponsor {5b}	Department of DefenseCongressionally Directed Medical Research Programs1077 Patchel StreetFort Detrick, Maryland, USA 21702-5024
Role of sponsor {5c}	The sponsor did not have a role in the design of this trial. The sponsor will not have a role in the collection, management, analysis, or interpretation of the data or any decisions to submit reports for publication.

## Introduction

### Background and rationale {6a}

Exposure to brain injury via blast or blunt mechanisms disrupts multiple sensorimotor systems simultaneously. Veterans from both the Gulf War and Operation Iraqi Freedom/Operation Enduring Freedom (OIF/OEF) campaigns in addition to active-duty service members report physical, sensory, cognitive, and behavioral/emotional changes [1–4]. Typically, symptoms related to these damaged systems recover within weeks, and resolution is often seen within 3 months [4]. However, a significant portion of the population of wounded soldiers suffer long-term functional consequences including visual deficits, postural and locomotor instabilities, disorientation, dizziness, sensitivity to visual and body motion, and an impaired ability to read. Many of these symptoms are overlooked in patients with polytrauma [5]. Earlier descriptions of such symptoms reported a third of service members (SM) exposed to blast trauma had combined visual and hearing impairment, termed dual sensory impairment [6, 7]. More recent evidence suggests that within the population of soldiers exposed to traumatic brain injury, a clinical pattern of damage to the auditory, visual, and vestibular sensorimotor systems has emerged, which has collectively been given the name multi-sensory impairment (MSI) [8, 9].

Nearly 20% of veterans diagnosed with the mild form of traumatic brain injury (mTBI) have MSI, as examined from a database of > 13,700 veterans [8, 10]. More than 350,000 US Gulf War era and OIF/OEF veterans with mTBI are suffering from MSI [10–12]. Among a variety of predictors (i.e., older age, female gender, posttraumatic stress disorder), having a prior history of mTBI was the most robust at predicting MSI. Among active-duty SM, 15–25% deployed to Iraq or Afghanistan sustained an mTBI during their tour, though other studies have reported a larger percentage (44%) [2–5]. In the US civilian population, MSI-related symptoms are also a common sequela of damage to the vestibular system and mTBI affecting approximately 300–500/100,000 population [13, 14]. Irrespective of the environment (military or civilian) or cause (mTBI or peripheral vestibular injury), the inner ear is commonly damaged when symptoms of MSI are experienced that involve both hearing (conductive, sensorineural, or mixed) and vestibular loss [15, 16].

### Objectives {7}

The four aims of the INVENT VPT Trial are to:

- Aim I: compare gaze and gait stability outcome measures between a novel (incremental velocity error (IVE)) and standard of care vestibular rehabilitation (VPT) intervention
- Aim II: compare the unique effect of gaze stability training only (delivered via IVE or VPT) on posture and gait outcome measures
- Aim III: investigate the optimal frequency of gaze stability exercises taking into account the burden on the patient and current best evidence
- Aim IV: characterize inter-trial correlations of the gain of the vestibulo-ocular reflex (VOR), which may predict the likelihood of vestibular adaptation in both mTBI and civilians with unilateral vestibular hypofunction (UVH)

### Trial design {8}

INVENT VPT is a randomized controlled crossover trial in which participants are allocated in a 1:1 ratio to initially receive the current standard of care vestibular rehabilitation method based on recent clinical practice guidelines (VPT) or incremental vestibular adaptation training (IVE). The novel incremental velocity error (IVE) method of vestibular training uses a device developed from laboratory studies to increase/normalize the gain/response of the vestibulo-ocular reflex (VOR).

Aims I–III include a crossover design with a washout period—though the duration of the aims and washout periods vary. After the washout period, participants will then receive IVE (if randomized to initially receive VPT) or VPT (if randomized to initially receive IVE). The outcome measures recorded after the washout on the 1st return visit are incorporated into the respective training for both arms. For example, participants are given balance exercises depending on their functional ability, which may change if the arm provides such an effect thereby minimizing any carryover effect during washout. Each participant recruited for the INVENT VPT Trial will receive a home exercise program to complete and be seen weekly in the clinic for exercise progression. The home exercises consist of gaze stability training (IVE or VPT) and gait/balance exercises. Exercises (gaze stability and gait/balance exercises) within the VPT intervention are developed based on the clinical practice guidelines [17]. Participants receiving IVE will use the IVE device and perform the same gait/balance exercises as participants receiving VPT. Each aim will include newly recruited participants so participants from one aim will not be eligible to participate in subsequent aims. Aim IV is an exploratory aim using data from all three prior aims. There is no additional data collection for aim IV.

## Methods: participants, interventions, and outcomes

### Study setting {9}

INVENT VPT data collection will occur at two clinical centers in the USA. Active-duty SM will be recruited from patients attending the Fort Belvoir Community Hospital (FBCH) in Fort Belvoir, VA. Civilians will be recruited among patients at the Johns Hopkins University (JHU) School of Medicine Outpatient Center in Baltimore, MD.

### Eligibility criteria {10}

The following are the inclusion criteria:

- Age 18–83 years old
- Service members with mTBI and civilian patients with vestibular hypofunction, both of which must report vestibular symptoms (i.e., dizziness, imbalance)
- Visual acuity with correction is ≥ 70 Early Treatment Diabetic Retinopathy Study (ETDRS) letter score (right and left eye)
- Visual acuity without correction is ≥ 35 ETDRS letter score (right and left eye)
- Participant has been diagnosed with a mild traumatic brain injury (FBCH site)
- Participant has been diagnosed with vestibular hypofunction (JHU site)
- Systolic blood pressure is ≤ 200 mmHg and/or diastolic is ≤ 110 mmHg at rest

The following are the exclusion criteria:

- Any person with a self-reported history of significant ophthalmic, neuromuscular, cardiovascular (except hypertension), renal/electrolyte, and Meniere’s disease
- Persons with uncontrolled severe hypertension (systolic blood pressure of > 200 mmHg and/or a diastolic blood pressure of > 110 mmHg at rest)
- Persons with a recent history of alcohol and/or drug abuse within the past 6 months
- Persons with benign paroxysmal positional vertigo
- Cervical spine active range of motion < 45°

### Who will take informed consent? {26a}

Certified study coordinators at each clinical center will introduce the trial to potential participants and will provide a brochure and consent document that describes the study procedures. Initial discussions will occur between the study coordinator and the potential participant and study investigators will also be available to discuss INVENT VPT aims and procedures.

### Additional consent provisions for collection and use of participant data and biological specimens {26b}

Participants are notified at the time of consent for the trial that their data may be shared even after the study ends with research collaborators, the sponsor, or through government or other databases or repositories. Data will be shared without personal identifiers. No ancillary studies are planned for the INVENT VPT Trial.

### Interventions

#### Explanation for the choice of comparators {6b}

##### IVE

**Fig. 1:**
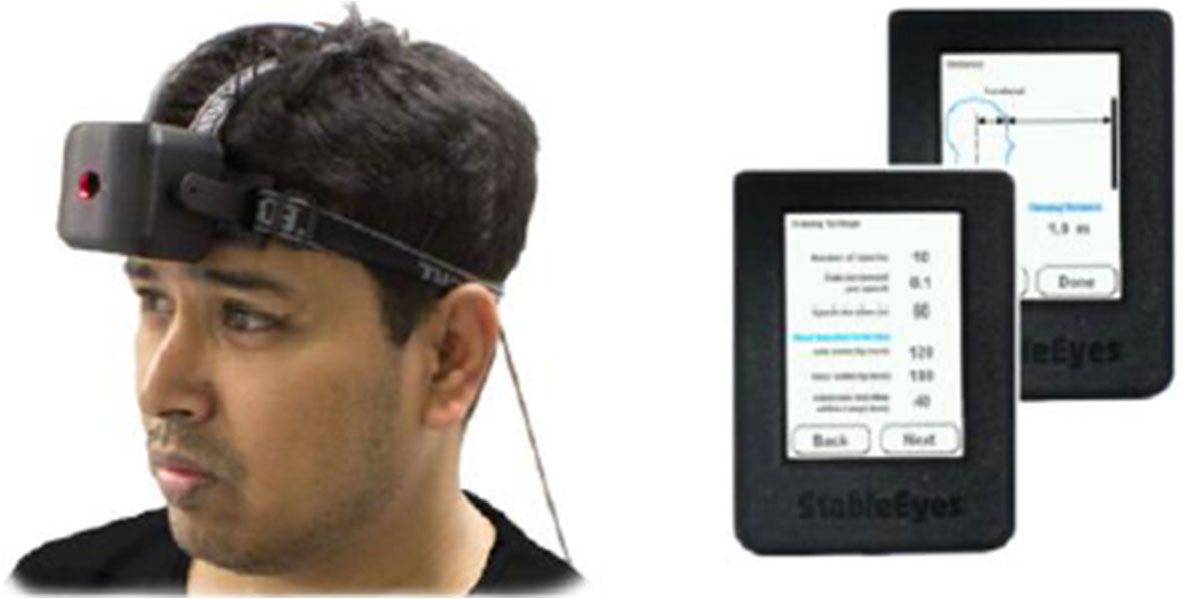
A participant wearing the head unit of the IVE training device. The base unit illustrates the configuration screens

The IVE experimental comparator incorporates a portable device with micro-electronics that allows the training to be unassisted and performed at home. The device consists of a head unit and a base unit (Fig. 1). The head unit contains inertial sensors to measure the instantaneous 3-D orientation of the head in space at 250 Hz and an integrated circuit mirror to dynamically control the position of a laser target in space. The base unit consists of a touch screen interface that allows users to calibrate and set the device, in addition to recording compliance [18].

##### VPT

The VPT control group will participate in gaze stability training per the current standard of care, which involves a set of active head rotation exercises to be performed daily [17].

#### Intervention description {11a}

**Fig. 2:**
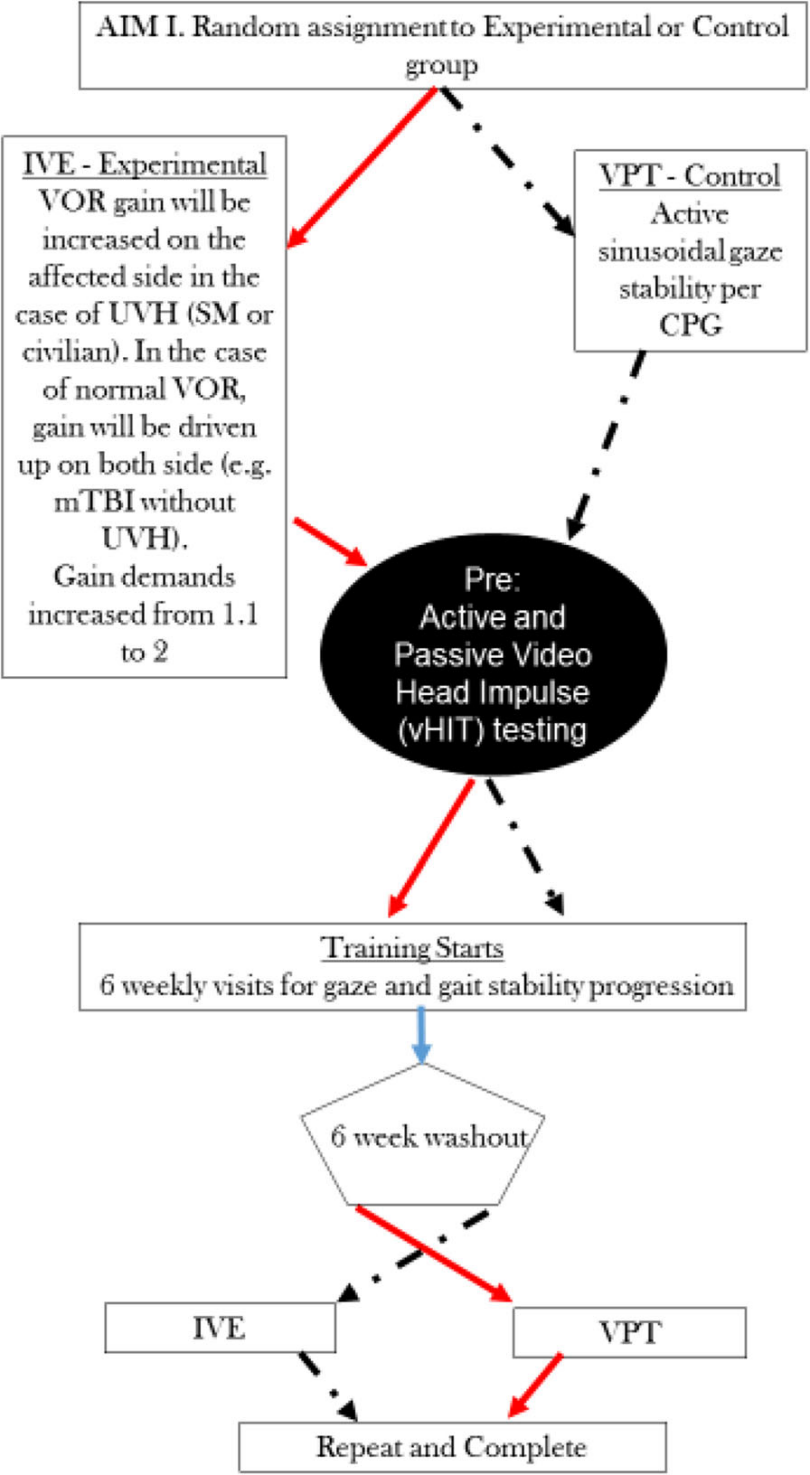
Algorithm of aim I comparing IVE with VPT, considering 6-week washout and crossover design. Both groups get gait/balance exercises from the beginning. CPG, clinical practice guidelines [17]

For aim I, we will randomize participants to the control (VPT) or experimental (IVE) group. For the following 6 weeks, participants in each group will be seen weekly for the progression of their gaze and gait stability exercises. Gaze stability will be for a total of 15 min per day for each group. After 6 weeks, participants will enter the washout stage for 6 weeks and not perform any explicit rehabilitation. Following the washout, participants will cross over into the other treatment group and complete another 6 weeks of IVE or VPT rehabilitation (Fig. 2).

For aims II and III, we will also randomize participants initially to the IVE or VPT group with a washout period and crossover to the other intervention. As with aim I, each group from aim II and aim III will participate in rehabilitation at home and will visit the clinic once per week for the modification of the gaze stability exercises. However, participants in aims II and III will have a shortened participation and washout. The duration for participation in aims II and III is reduced from aim I based on our desire to determine ideal dosing for gaze stability training considering burden and compliance. Additionally, the expectation for most patients undergoing vestibular rehabilitation is that 6 weeks is a typical time to expect change (per currently clinical practice guidelines (CPG)) [17].

**Fig. 3:**
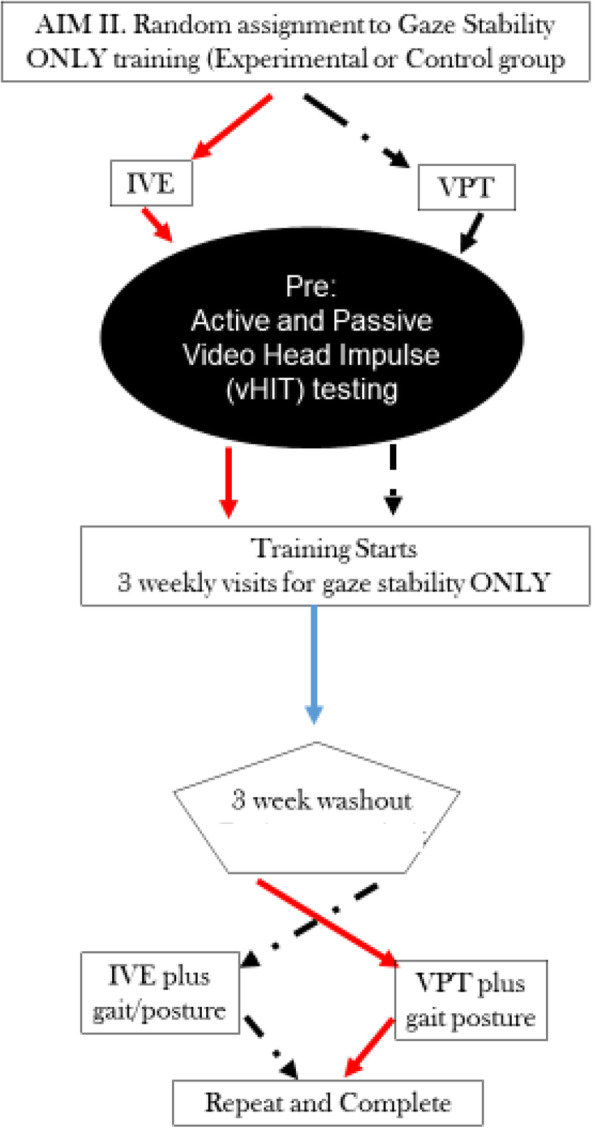
Algorithm of aim II examining how training gaze stability alone impacts improvement considering 3-week washout and crossover design. Gait/balance exercises do not start until after the washout

In aim II, neither IVE nor VPT groups will complete their balance and gait exercises until the washout period begins (Fig. 3). Each group will perform daily gaze stability exercises: VPT control group will follow the recommended CPG (15 min), and members in the IVE group will use the IVE device (15 min). Participants will perform the gaze stability exercises only for 3 weeks, before entering the washout stage for 3 weeks and not perform any explicit rehabilitation. Once they enter washout, they will begin gait/balance home exercises. Once the washout is complete, they will cross over into the other group for gaze stability training and continue with gait/balance exercise and progression (Fig. 3).

For aim III, we seek to identify a minimum effective dose of gaze stability training. To do this, IVE and VPT participants will each complete their respective gaze stability training every other day. Both groups will complete the same gait/balance exercises from the start of the aim. Excluding the duration, the flow of aim III (Fig. 3) is similar to aim I.

For all aims, the intensity of the exercise program will be determined at the baseline visit based on the validated findings of the Dizziness Handicap Inventory, gait speed, and functional gait assessment (Table 1). Scores on each of these assessments will be summed for a composite impairment score. The composite score will inform the development of the exercise program to address the severity of impairment.

**Table 1:** Schedule of enrollment and assessments in the INVENT VPT Trial

Study period				
Enrollment/baseline and randomization	Interventions	Follow-up assessments		
		vHIT	Participative measures	Impairment measures
**Eligibility screen** **Informed consent** One-time VOR characterization • Videonystagmography • Ocular and cervical vestibular evoked myogenic potential • Rotary chair • Video head impulse test • Audiogram • Clinical vestibular exam • Static visual acuity • Seated blood pressure **Randomization** • Treatment intensity ♦ Dizziness Handicap Inventory ♦ Gait speed ♦ Functional gait assessment	**Aim I** IVE-VPT VPT-IVE 6-week washout	Weeks 1–6 Weeks 13–18 6 months	Weeks 1, 4, 6 Weeks 13, 16, 18 6 months	Weeks 1, 4, 6 Weeks 13, 16, 18 6 months
	**Aim II** IVE-VPT VPT-IVE 3-week washout	Weeks 1–3 Weeks 7–9 6 months	Weeks 1, 3 Weeks 7, 9 6 months	Weeks 1, 3 Weeks 7, 9 6 months
	**Aim III** IVE-VPT VPT-IVE 3-week washout	Weeks 1–3 Weeks 7–9 6 months	Weeks 1, 3 Weeks 7, 9 6 months	Weeks 1, 3 Weeks 7, 9 6 months

#### Criteria for discontinuing or modifying allocated interventions {11b}

Participants reporting neck pain would be examined during a clinical visit to assess whether the exercises are being performed as described in the protocol. If the participant develops severe neck pain (sudden severe sustained lasting 24 h) that does not resolve within 48 h, the participant would be referred to their treating physician for follow-up care. Medication will be prescribed for symptomatic relief as deemed appropriate by the participant’s treating physician. If the severe neck pain persists 3 days after treatment, the intervention would be discontinued. Modifications to interventions or discontinuation of study interventions will be documented on a protocol deviation form.

#### Strategies to improve adherence to interventions {11c}

In addition to the exercise program, we will provide a weekly paper exercise completion grid for participants to record exercise completion. The exercise completion grid will also serve as a reminder to complete the exercises. We will also use the information provided in the exercise grid to track compliance. We will provide the participant with a new exercise grid at each visit. We have used this method with success to assist the completion of weekly exercises and track compliance in prior studies [19].

#### Relevant concomitant care permitted or prohibited during the trial {11d}

There are no prohibited interventions, although we will request that participants not enroll in any other clinical trial comparing balance therapies for the duration of their INVENT VPT participation.

#### Provisions for post-trial care {30}

Medical care will be provided to participants that may experience harm from participating in the INVENT VPT Trial. The cost for any treatment or hospital care that may be received because of a study-related injury will be billed to the participant if not covered by a health insurer. The participant does not waive their rights to seek compensation for any injuries received because of trial participation.

### Outcomes {12}

The primary outcome measure will be VOR gain (eye velocity/head velocity) as measured using the video-head impulse test (vHIT). The vHIT affords us the ability to identify other mechanisms of gaze stability (i.e., compensatory saccade); thus, we will also capture metrics related to compensatory saccade frequency and latency. We will assess inter-trial correlations for both VOR gain and compensatory saccade latency. Secondary outcome measures include compensatory saccade metrics and those that capture data in the participative and impairment domains. Participative domain measures include the Dizziness Handicap Inventory (DHI), the Activities-Specific Balance Confidence Scale (ABC), the Neurobehavioral Symptom Inventory (NSI), and the Patient Global Impression of Change (PGIC). Finally, a one-time characterization of hearing and vestibular function will be obtained at enrollment.

#### Participative measures

The DHI measures a participant’s perception of how the dizziness impacts their life. The index considers the emotional, physical, and functional aspects of their quality of life. Clinical significance is defined as a decrease in the DHI of either 18 points or 42% from the pre-treatment level [19]. VPT is known to reduce DHI score [20–22].

The ABC is a self-reported instrument that asks participants to rate their confidence performing 16 activities of daily living [23, 24]. Scores range from 0 indicating no confidence to 100 indicating complete confidence in the participant’s ability to perform the task without losing their balance. Scores < 67% indicate a risk for falling and accurately classify people who fall 84% of the time [25]. The ABC has excellent test-retest reliability (*r* = 0.92), and VPT is known to improve the ABC score [26].

The NSI collects data on the symptoms experienced after an mTBI. We will follow the Department of Defense’s suggestion that an individual experiencing a minimum of 20% score improvement from baseline is considered significant [27].

The PGIC is a commonly used method to quantify the amount of improvement the patient believes has occurred since beginning treatment (i.e., intervention). The PGIC asks one question that is rated on a 7-point Likert scale. PGIC values of 6 or more correlate best with actual change [28].

#### Impairment measures

Impairment domain measures include the dynamic visual acuity test (DVA), the instrumented stand and walk test (ISAW), vertical and torsional alignment nulling (VAN and TAN), and the following clinical measures that we will instrument using inertial measurement units (9 degrees of freedom sensors)

- Functional gait assessment (FGA)
- Gait speed
- Tandem walk
- Modified clinical test for sensory integration of balance (mCTSIB)

The DVA test will measure visual acuity during active impulse head rotations. Participants will wear a headband with a rate sensor attached that triggers a flashing letter once head velocity crosses a predetermined threshold. Participants will generate a single head rotation to the right and left (impulses, separately) with performance being assessed from the size of the smallest letter the participant can identify [19].

The ISAW uses wireless sensors to measure a 30-s stand, 7-m walk, followed by a 180° turn. ISAW can distinguish soldiers with mTBI from healthy controls by their longer duration to turn (*p* < 0.001), their increased number of steps to complete a turn (*p* < 0.001), and their decreased peak velocities during the turn (*p* = 0.003) [28].

During vertical alignment nulling and torsional alignment nulling (VAN, TAN), the participant views one red and one blue line on the tablet screen through color-matched red and blue filters. The tests measure the perception of ocular alignment and are reliable and valid in conditions of otolith dysfunction.

The FGA is an 8-item scale that was developed to determine fall risk in older adults. Participants are scored on a 4-level ordinal score while they perform various ambulation tasks. Scores < 20 indicate an increased risk of falling in older adults.

The Patrol Exertion Multitask Test (PEMT) involves significant cognitive demands of situational awareness, memory, and decision-making under the physical stress of moderate exertion. The PEMT is a 12-min test where participants view a virtual patrolling scenario while responding to intermittent and unpredictable reaction timing cues. During this, they report visual clarity and perceived exertion. The PEMT has excellent interrater reliability for the assessment of cognitive (ICC 0.97), visual (ICC 0.99), and exertional (ICC 0.98) domains and can distinguish active-duty service members (SM) with mTBI from healthy control service members based on scores of visual clarity and reaction time [29].

The Modified Clinical Test of Sensory Interaction on Balance (mCTSIB) tasks participants to stand quietly (arms folded) while sway and time are measured under four conditions: (1) eyes open firm surface, (2) eyes closed firm surface, (3) eyes open unstable surface (foam), and (4) eyes closed unstable surface (foam). The best possible timed scores are 30 s for each item or 120 s total score.

### Participant timeline {13}

Table 1 illustrates the enrollment, interventions, and assessments of the INVENT VPT Trial. The primary outcome is VOR gain as measured by the vHIT. Secondary outcomes include participative and impairment measures. During the enrollment/baseline visit, participants will receive an initial VOR characterization and complete assessments for determining exercise treatment intensity. Participants will be randomized to IVE washout period-VPT or VPT-washout period-IVE. The washout period varies as do the timing of follow-up assessments by aim.

### Sample size {14}

We have based our estimate of sample size on data published in healthy adults (21–58 years old) using the IVE paradigm, whose age reflects our target patient population [30]. In that study, active VOR gain pre-adaptation was 0.92 ± 0.18 and active VOR gain post adaptation was 1.11 ± 0.22. Therefore, using the mean difference between pre- and post-adaptation of 0.19 with a standard deviation of 0.18, we would need a minimum of 9 participants to be able to reject the null hypothesis with a probability (power) of 0.8, presuming *α* < 0.05 (PS Power and Sample Size Calculations, version 3.1.2). Presuming a 25% attrition, we will recruit at least 12 participants for each of the IVE-VPT and VPT-IVE patient cohorts (aims). We aim to enrol 24 service members at the FBCH site and 24 civilians at the JHU Outpatient Center for each of aims I–III.

### Recruitment {15}

Participants will be recruited via a combination of methods that include a chart review, in-person recruitment during clinic visits, and queries garnered using flyers posted in clinics.

### Recruitment at the FBCH

In 2017, 226 SM with mTBI were evaluated for mTBI at FBCH. In the past, researchers from FBCH have had success recruiting 2–3 SM/month with mTBI for participation in studies (personal communication, Stephanie Beauregard PT). We intend to screen ~ 10 patients per month for the INVENT VPT, with the intention of enrolling 2–3 participants per month.

### Recruitment at Johns Hopkins University School of Medicine (JHU)

At the JHU Outpatient Center, the principal investigator (PI) of the INVENT VPT routinely sees approximately 20 patients per month with dizziness and balance disorders, ~ 120/year. Of those 20 patients per month, roughly 20% have a peripheral vestibular hypofunction. Therefore, we intend to screen a minimum of 16 patients per month to obtain the required number of enrolled participants with vestibular hypofunction, over the duration of the study.

## Assignment of interventions: allocation

### Sequence generation {16a}

Random assignments to VPT-IVE or IVE-VPT will be allocated on a 1:1 ratio via a computer-generated randomization schedule stratified by the clinical center and utilizing random permuted blocks of random sizes. Block sizes are not disclosed.

### Concealment mechanism {16b}

Participants will be assigned to VPT-IVE or IVE-VPT via an online central randomization program in REDCap that is maintained by the Data Coordinating Center. The random assignment is released to the study coordinator within the REDCap randomization program after the baseline study visit has been completed and the participant has been confirmed as eligible for the trial. The random assignments for the INVENT VPT will be maintained by the Data Coordinating Center and will not be accessible to the study investigators and clinical center personnel.

### Implementation {16c}

Patients who provide informed consent and are deemed eligible for the INVENT VPT Trial will be randomized to IVE-VPT or VPT-IVE. The allocation sequence will be generated by the Data Coordinating Center. Study coordinators will enroll participants after confirming eligibility via a screening form. The coordinator will enter specific baseline assessment data into a computerized randomization program generated in REDCap, and the assignment will be provided after these data are entered and confirmed by the study coordinator. The sequence of allocations will not be disclosed to the study site and will be maintained by the Data Coordinating Center.

## Assignment of interventions: blinding

### Who will be blinded {17a}

Due to the nature of the interventions, study participants and the research physical therapists administering the interventions are unable to be blinded/masked to treatment assignment. The study coordinator is unmasked to treatment assignment as they are involved in the randomization process. The research physical therapist will not be involved in the measurement of any trial outcomes. The research audiologist will be masked to treatment assignment and will conduct the auditory and vestibular function tests. A member of the research team other than the research physical therapist/audiologist will conduct the measurement of all trial outcomes. Participants and unmasked research personnel will be advised not to share treatment assignments during the follow-up visits. INVENT VPT study forms that disclose treatment allocation will be entered by the study coordinator, and these study forms will be kept separate from the participant’s medical records.

### Procedure for unblinding if needed {17b}

If unmasking of the research technologist is deemed necessary, the clinical center investigator will notify the Data Coordinating Center and note the reason for unmasking on the data collection form. Investigators will be encouraged to maintain masking unless there is reason to believe that unmasking the research technologist is deemed to be in the best interest of the participant’s safety.

## Data collection and management

### Plans for assessment and collection of outcomes {18a}

Plans for assessment and collection of outcomes are discussed in the “Outcomes {12}” section.

#### Training and certification of study personnel

All study personnel responsible for recruiting, enrolling, collecting, and entering INVENT VPT data will receive role-specific training on the study procedures to include virtual and in-person training sessions, written examinations, and direct observation sessions. Study procedures, including the follow-up schedule, data collection, completion of the data collection forms, and the data entry and data quality query protocol will be discussed during live and recorded presentations. Personnel who satisfactorily complete all INVENT VPT training and receive passing scores on written examinations/direct observation sessions will be certified to participate in the INVENT VPT and will receive a certification ID to be used as a personnel identifier on all study documents.

### Plans to promote participant retention and complete follow-up {18b}

Study coordinators will develop a schedule for contacting participants by email, telephone, or text message (depending on the participant’s preference for contact) to remind them of upcoming study visits and to remind them to continue their study-assigned rehabilitation and other related procedures while at home.

We will also share the results of testing, including the video head impulse test reflecting the integrity of the vestibulo-ocular reflex measures, with participants at the conclusion of INVENT VPT Trial participation and include comparisons with age-matched controls.

### Data management {19}

Clinical center personnel who have been certified for INVENT VPT data entry will be provided access to the REDCap data system that will be used for data entry and access to the data for their center only. We have designed a double data entry system with an automated range and missing data checks upon entry. Inconsistencies will be further checked after entry via a series of programs that are designed to detect specific within-form and cross-form errors. Data quality query reports will be distributed biweekly to each clinical center for resolution. The study coordinator will be responsible for making corrections to the data system based on the queries that are distributed. The REDCap data system records all entries to maintain an audit trial of changes to the data system. Each clinical center will be responsible for responding to all queries and returning their responses to the Data Coordinating Center (DCC) for review.

### Confidentiality {27}

Participants who agree to be screened for eligibility will be assigned a study ID that will be used on all data collection forms. All data will be associated with the anonymous number and personal information will no longer be used. All data are stored on secured password-protected media. There will be a master document that links identifiable information with the anonymous identifier. This master document will only be accessible to the study coordinator. All participant study documents will be stored in a locked cabinet in a location with controlled access.

### Plans for collection, laboratory evaluation, and storage of biological specimens for genetic or molecular analysis in this trial/future use {33}

Biological specimens are not collected in this trial.

## Statistical methods

### Statistical methods for primary and secondary outcomes {20a}

The primary outcome for determining treatment efficacy is VOR gain, which is measured weekly for each subject in each intervention period. To verify randomization between sequences, baseline demographics and characteristics of vestibular injury will be compared across arms. The primary analysis will follow the intent to treat principle, where patient outcomes are analyzed following how they were randomized and not adjusted or excluded based on adherence to assigned treatment. To determine the efficacy for the primary outcome, a generalized linear mixed model will be used with VOR gain as the dependent factor [31]. This model will be used to compare VOR gain between interventions and to estimate period and carryover effects. With substantial washout periods for each aim, we do not expect there to be substantial differences in the carryover effects between intervention sequences. We will verify there are no differences in the carryover effects by testing for an interaction between treatment and time. If this interaction is statistically significant at *α =* 0.05, we will focus our analysis on patient outcomes in the first period, and disregard outcomes in the following period. We will adjust this model for site as well as demographic and vestibular injury-related factors that are imbalanced between arms at baseline. All statistical tests will be conducted as two-sided for an *α* of 0.05.

For secondary analyses, we will examine the compensatory saccade metrics, which are also measured at each weekly visit similarly to VOR gain. Similar to our analysis of the primary outcome, we will examine this secondary outcome with a generalized linear mixed model, estimating carryover effects by testing for an interaction between period and intervention. We will also adjust these comparisons of VOR gain and compensatory saccade metrics for site and for factors that are imbalanced at baseline. Analyses with greater than 25% loss to follow-up or incompleteness for other reasons will use multiple imputation [31]. All analyses will be conducted using the SAS software version 9.4 (SAS Institute Inc., Cary, NC, USA).

### Interim analyses {21b}

No interim analyses are planned for the INVENT VPT Trial.

### Methods for additional analyses (e.g., subgroup analyses) {20b}

This is addressed in the “Statistical methods for primary and secondary outcomes {20a}” section.

### Methods in analysis to handle protocol non-adherence and any statistical methods to handle missing data {20c}

This is addressed in the statistical methods section.

### Plans to give access to the full protocol, participant-level data, and statistical code {31c}

Investigators engaged in the Department of Defense-sponsored research must share participant-level data and the study protocol to the Federal Interagency Traumatic Brain Injury Research Informatics System (FITBIR). De-identified INVENT VPT data will also be available upon request to the principal investigator.

### Oversight and monitoring

#### Composition of the coordinating center and trial steering committee {5d}

The INVENT VPT Data Coordinating Center (DCC) is responsible for assuring that the proposed study design and methods are statistically sound, that the investigators and study personnel adhere to the protocol, that valid and reliable data are collected and integrated, and that the accumulated data are summarized and reported as required to monitor study progress and promote and assess data quality. The DCC is composed of a director, data programmer, and biostatistician. A trial steering committee has not been formed for this trial.

Specific tasks are given by phase of the INVENT VPT Trial. Some of the operations at the DCC are designed to facilitate operations at the clinical centers.

#### Initial design phase

DCC responsibilities during this period are as follows:

- To assist with the development of the study design and to estimate sample size requirements
- To outline the data collection methods, data management methods and operations, and plans for data reporting
- To outline quality assurance and monitoring procedures
- To draft, critique, and revise major portions of the manual of procedures and assure adherence to the protocol
- To evaluate currently available hardware and software for data collection, management, and analysis
- To design the training session for the INVENT VPT

#### Protocol refinement and implementation phase

This phase begins when funding is awarded and ends when patient enrollment is initiated or shortly thereafter. DCC responsibilities during this period are as follows:

- To test and refine data collection methods in REDCap
- To complete assigned portions of the manual of procedures and edit for consistency and ease of reference
- To implement the central data management system and test procedures and operations, including tasks such as acquisition, installation, and implementation of necessary hardware and software; writing data definition tables; and editing of tables for data items to be collected
- To develop materials for training and certifying clinical center staff in INVENT procedures
- To design aids to assist with the management of all INVENT centers, including reports to clinical centers
- To update INVENT data collection forms and distribute electronic copies to the clinical centers
- To undertake site initiation visits and to ensure timely startup

#### Enrollment and follow-up phases

The following are the data management responsibilities:

- To receive clinical data from INVENT VPT clinical center staff via REDCap
- To edit all data for completeness, accuracy, and consistency and to resolve anomalous data with clinical center staff
- To revise data entry screens as required
- To prepare periodic reports concerning enrollments, completeness of scheduled follow-up interviews, and database quality

The following are the data analysis and reporting responsibilities:

- To employ appropriate statistical methods for analysis and summarization of accumulated data based on the recommendations of the study biostatistician
- To undertake the final data analyses for INVENT VPT primary manuscripts
- To maintain the data files in a secure manner to assure their integrity
- To back up data files to assure that data are not lost
- To maintain a program of regular monitoring of the quality of the INVENT VPT database

#### Composition of the data monitoring committee, its role, and reporting structure {21a}

The interventions included in the INVENT VPT are considered minimal risk so a data monitoring committee will not be included in this trial. An independent medical monitor will review all adverse events that are reported during the conduct of the INVENT VPT Trial and assess the potential that the event may be attributed to the study interventions or participation in the trial. The medical monitor’s findings will be reported to the IRB.

#### Adverse event reporting and harms {22}

Some of the tests may cause symptoms of neck pain, dizziness, nausea, and/or motion sickness. The participant will be asked to report any of these symptoms to the investigator. The participant or the investigator may stop testing at any time. These symptoms usually resolve once testing has stopped. Infrequently, motion sickness symptoms may persist for as long as a few hours.

To minimize the risk of neck strain, severe neck pain, or other cervical injuries due to head-on-body movements, all such movements are first self-generated by the participants. Passively received (i.e., delivered by the experimenter) head movements are kept within the participant’s demonstrated range of self-generated head movements. In keeping with standard clinical practice, participants with known cervical spine instability are not subjected to tests requiring rapid head-on-body movements. To minimize the risk of skin irritation, the skin of the neck will be examined for irritation before we apply electrodes for vestibular evoked myogenic potential testing.

Balance and gait testing have a small risk of falling. To ensure that participants do not fall, testing is conducted with investigators or clinical support staff placed around them to assist as needed. Medical risks, listing all procedures, their major and minor risks, and expected frequency are always conveyed to the participants.

Any adverse events will be recorded on an adverse event form and reported within 24 h to the principal investigator. The principal investigator will notify the IRB by the next business day.

#### Frequency and plans for auditing trial conduct {23}

Onsite and remote data monitoring will be completed via an export of key data from the RedCap database. Data monitoring of key data points will be completed by the DCC. The responsibilities for remote data monitoring at the DCC are as follows:

- To develop and test a SAS program to monitor incoming data for inconsistencies based on cross form, within form, cross visit, range, consistency, and expected data entry checks
- To execute the SAS program that monitors incoming data for inconsistencies on a weekly basis during data collection
- To distribute data quality queries to Clinical Coordinators biweekly

Data entered into the REDCap system by the Clinical Centers will also be monitored for out-of-range values, inconsistent data, and double data entry inconsistency and completeness.

Periodic in-person site visits are necessary to ensure that there is standardization of procedures, that personnel have been trained adequately, that the clinical facilities meet standards, and that participants and their data are being managed as specified in the protocol. A standard agenda is used for monitoring visits. All sites are visited within the first 3 months of the initiation of participant recruitment and then again at 6 months after the commencement of participant recruitment. Other clinical center monitoring visits shall be conducted on an as-needed basis.

General areas of review during the site visit include the following:

- Clinic staff, facilities, and equipment
- Flow of participants through the clinic
- Up-to-date study documentation including the manual of procedures, data collection forms, and documentation confirming reports of serious adverse events to the local IRB and other regulatory documents
- Review of signed consent forms for 100% of participants
- Review of a sample (approximately 10%) of data collection forms for comparison with data in the REDCap database and source documents
- Observation of the study coordinator during a participant visit, if possible
- Storage and access to study participant files, including proper storage of signed consent forms and handling of data quality queries
- Discussion of individual participants with compliance problems that may be alleviated by the study coordinator or other study personnel
- Meeting with the principal investigator of the clinic to discuss any areas of concern

The PI, study coordinator, and site co-investigators receive a detailed report of the findings within 7 business days. Recommendations for remedial actions with a timeline for resolution are included, if necessary. The monitor will also inform the study PI of any remedial actions recommended within 7 business days of the site visit and will submit a subsequent report to the PI summarizing resolution of all deficiencies within 14 business days of final resolution by the clinical center. Remote monitoring will be independent from the study investigators and the Department of Defense.

#### Plans for communicating important protocol amendments to relevant parties (e.g., trial participants, ethical committees) {25}

Protocol amendments, including any modifications to the eligibility criteria, study design, recruitment procedures, or analysis plan will be documented in a revised protocol with a new version number and date. These modifications will be submitted to the IRB and approved prior to distribution to the clinical centers. All protocol modifications, including those that do not significantly impact the conduct of the study, will be communicated to the INVENT VPT investigators and personnel via a policy and procedures memorandum.

#### Dissemination plans {31a}

It is expected that the PI of the INVENT VPT Trial will be responsible for the dissemination of the INVENT study methods and findings in appropriate locations of visibility (talks, journals). As the data collection develops and statistics are applied, it will be the PI’s responsibility to discuss authorship and order of authorship with the appropriate study team members. It is likely that many of the eventual manuscripts will include references to the INVENT VPT study team participants, rather than listing each individual as an author. Ultimately, a decision on authorship will be made via study team discussion and the final decision by the PI.

## Discussion

### Trial status

Protocol version number: 1.0

Protocol date: 15 October 2020

At the time that this protocol was initially submitted to *Trials*, recruitment had not yet commenced. Recruitment has since commenced in October 2021.

## Data Availability

The Data Coordinating Center will be responsible for executing the data sharing process for internal INVENT VPT investigators. Study investigators will have access to the final data sets, and password-protected data will be provided upon request. Data will be stripped of identifying information before distribution.

## Abbreviations

ABC: Activities-Specific Balance Confidence Scale
CPG: Clinical practice guidelines
DCC: Data Coordinating Center
DHI: Dizziness Handicap Inventory
DVA: Dynamic visual acuity
ETDRS: Early Treatment Diabetic Retinopathy Study
FITBIR: Federal Interagency Traumatic Brain Injury Research Informatics System
FBCH: Fort Belvoir Community Hospital
FGA: Functional gait assessment
IVE: Incremental velocity error
INVENT VPT: Incremental Velocity Error as a New Treatment in Vestibular Rehabilitation
ISAW: Instrumented stand and walk test
JHU: Johns Hopkins University
mTBI: Mild traumatic brain injury
mCTSIB: Modified clinical test for sensory integration of balance
MSI: Multisensory impairment
NSI: Neurobehavioral Symptom Inventory
OEF: Operation Enduring Freedom
OIF: Operation Iraqi Freedom
PGIC: Patient Global Impression of Change
PEMT: Patrol exertion multitask test
PI: Principal investigator
SM: Service members
TAN: Torsional alignment nulling
UVH: Unilateral vestibular hypofunction
VAN: Vertical alignment nulling
VPT: Vestibular rehabilitation
VOR: Vestibulo-ocular reflex
vHIT: Video-head impulse test
